# QTL mapping of modelled metabolic fluxes reveals gene variants impacting yeast central carbon metabolism

**DOI:** 10.1038/s41598-020-57857-3

**Published:** 2020-02-07

**Authors:** Matthias Eder, Thibault Nidelet, Isabelle Sanchez, Carole Camarasa, Jean-Luc Legras, Sylvie Dequin

**Affiliations:** 1 SPO, Univ Montpellier, INRAE, Montpellier SupAgro, Montpellier, France; 2 MISTEA, INRAE, Montpellier SupAgro, Montpellier, France

**Keywords:** Computational models, Quantitative trait

## Abstract

The yeast *Saccharomyces cerevisiae* is an attractive industrial microorganism for the production of foods and beverages as well as for various bulk and fine chemicals, such as biofuels or fragrances. Building blocks for these biosyntheses are intermediates of yeast central carbon metabolism (CCM), whose intracellular availability depends on balanced single reactions that form metabolic fluxes. Therefore, efficient product biosynthesis is influenced by the distribution of these fluxes. We recently demonstrated great variations in CCM fluxes between yeast strains of different origins. However, we have limited understanding of flux modulation and the genetic basis of flux variations. In this study, we investigated the potential of quantitative trait locus (QTL) mapping to elucidate genetic variations responsible for differences in metabolic flux distributions (fQTL). Intracellular metabolic fluxes were estimated by constraint-based modelling and used as quantitative phenotypes, and differences in fluxes were linked to genomic variations. Using this approach, we detected four fQTLs that influence metabolic pathways. The molecular dissection of these QTLs revealed two allelic gene variants, *PDB1* and *VID30*, contributing to flux distribution. The elucidation of genetic determinants influencing metabolic fluxes, as reported here for the first time, creates new opportunities for the development of strains with optimized metabolite profiles for various applications.

## Introduction

The yeast *Saccharomyces cerevisiae* has been used for millennia for the production of various fermented foods and beverages^1^. In modern times, yeast has become popular in new applications, ranging from the biosynthesis of ethanol (for biofuels) and other raw materials (for chemical syntheses) to the production of fine chemicals used as fuel additives, flavours and fragrances, or medical components^2^. Advances in metabolic engineering are constantly expanding the range of yeast’s applications.

Many phenotypic traits of *S. cerevisiae* relevant to industrial processes are dependent on the functional and regulatory properties of central carbon metabolism (CCM)^3^. The yeast metabolic network that involves a large number of intracellular reactions is highly conserved and has evolved to be organized as a bowtie structure, meaning that all carbon sources are converted to 12 different precursor metabolites, which are then used by the cell for the biosynthesis of macromolecules that compose cellular biomass^4,5^. In addition, these precursors form the basis for the synthesis of extracellular metabolites, e.g., succinic acid, which is industrially produced by yeast fermentation as a building block for polymer production^6,7^. Furthermore, they serve as starting points for the heterologous production pathways of renewably produced fine chemicals^8,9^. The tight reduction of cellular carbon core metabolism to a small number of important metabolites results in a high carbon flux through these compounds^10^, controlled by a complex regulation on genetic (transcription, translation, protein modifications and protein-protein interactions) and metabolic levels. Therefore, understanding how metabolic flux distribution is controlled is a key requirement for increasing product biosynthesis by metabolic engineering^11^.

While metabolite turnover rates are difficult to determine experimentally, they can be estimated by modelling^12,13^. Commonly used constraint-based models (CBM) formulate metabolic networks as a stoichiometric matrix to predict intracellular fluxes through the application of experimental constraints on input and output fluxes. Depending on the network size and number of constraints, this approach, which is termed metabolic flux analysis (MFA), can be sufficient for estimating fluxes. However, in most cases, adding constraints on input and output data is not sufficient to estimate all fluxes of a network. One way to address this insufficiency is the ^13^C-MFA approach. It tracks ^13^C from labelled substrates across cellular metabolites with the aim of generating information to constrain and estimate intracellular fluxes. Another way is to apply the assumption that cellular functions of biochemical networks in a steady state are limited by physico-chemical constraints^14^. In this case, the flux balance analysis (FBA) approach chooses the best fitting solution through linear optimization out of a narrowed solution frame defined by the stoichiometric matrix of the CBM^15^. The outcome of this flux prediction depends on an applied objective function (maximization of ATP production, minimization of metabolic adjustment, or in most cases, maximization of biomass production)^16^.

To study how metabolic fluxes are modulated by genetic or environmental determinants, we previously used a combined ^13^C-MFA/FBA approach to estimate the intracellular fluxes of *S. cerevisiae* CCM in conditions of modified intracellular redox balance^17,18^. Another example of the application of FBA is the study of Quirós *et al*. (2013), which used a model developed by Vargas *et al*. (2011) to evaluate changes in yeast metabolism in high sugar must^19,20^. In both studies, glycolytic fluxes showed the least variation, whereas the fluxes of the pentose phosphate pathway (PPP) were highly variable. ^13^C-MFA, on the other hand, was used to study network robustness^21^ or the effects of deletion mutants^22^. The latter study demonstrated interesting links between networks, e.g., a positive correlation between the PPP and biomass yield.

In recent years, studies have generated vast amounts of information about the genotypic and phenotypic diversity of *S. cerevisiae* by comparison of growth parameters in different media^23–28^. Furthermore, several studies extended the characterization of diversity to a greater number of phenotypic traits, including life history traits and metabolic traits, showing that origin has a broad phenotypic impact and that part of these phenotypic differences can be explained by adaptation to the ecological constraints imposed by origin^29–31^.

Recently, we assessed the diversity of flux distributions between *S. cerevisiae* strains from different origins^32^, using the constraint-based model developed by Celton *et al*. (2012) to estimate CCM flux distributions between 43 strains grown under wine fermentation conditions^18^. The study showed a contrasting image regarding flux variability with quasi-constancy of glycolysis and ethanol synthesis on the one hand, but large variations in other fluxes, such as the PPP and acetaldehyde production, on the other hand. In addition, the fluxes’ multimodal distributions related to ecological origin revealed an association between genetic origin and flux phenotype^32^.

Results from flux analysis have been used for strain improvement (increased ethanol yield, optimization of metabolites production, …) by metabolic engineering based on flux predictions^33–38^. Therefore, more knowledge about the impact of genomic variation on metabolic flux distributions can potentially assist with the selection or improvement of strains for diverse applications, in the food and beverage industries as in the field of biotechnology.

Quantitative trait locus (QTL) mapping, which has been applied in numerous existing studies, has become an important approach to more deeply understand the genomic complexity of *S. cerevisiae* and decipher the impact of genomic variation on yeast complex traits^39^. This application includes investigations of genetic determinants influencing the formation of industrially relevant traits, which have led to the discovery of allelic variants accounting for variations in these traits^40–48^. In all these studies, the assessed traits were straightforward to quantify. However, difficulties remain in detecting QTLs for traits with small variations or those that are more challenging to determine, such as intracellular metabolic fluxes.

The possibility of using QTL mapping to decipher genomic variations impacting metabolic profiles (rather than those affecting single metabolites) would create ways to understand the mechanisms behind metabolic flux distributions and to engineer strains with superior metabolic properties for various applications. To achieve this aim, we phenotyped 130 meiotic F2-segregants from a cross of two *S. cerevisiae* yeast strains for their production of extracellular main metabolites during the exponential phase. We modelled the intracellular fluxes of yeast CCM by applying these experimentally determined metabolite concentrations to a constraint-based model^18^. Subsequently, we used these estimated fluxes as phenotypic data to perform QTL mapping on metabolic flux distributions. With this approach, we were able to detect four fQTLs that influenced various metabolic fluxes. By performing reciprocal hemizygosity analysis (RHA), we confirmed the robustness of the method by validating the role of two genes, *PDB1* and *VID30*, within two fQTLs. The allelic variants of these genes show different effects on the fluxes of glycolysis, ethanol synthesis, glycerol synthesis, the tricarboxylic acid (TCA) cycle and the excretion of TCA cycle metabolites.

## Results

### Phenotyping of strains

Fluxes of the CCM were predicted for all strains, using extracellular metabolite concentrations that were experimentally determined during the exponential growth stage (when cells are in a quasi-steady state). Because of the structure of the network, some fluxes are directly correlated. To assess for flux correlations, we analysed the relationships between steady-state reaction fluxes as described by Poolman *et al*. 2007^49^ in order to produce a correlation matrix (Supplementary Fig. S1). Based on the observed strong linkage between single reactions within main metabolic pathways, representative reactions were selected for these pathways to facilitate the following analyses (Table 1).

**Table 1:** Flux Selection. Selection of 20 fluxes that are representative of the main metabolic pathways.

Flux abbreviation*	Pathway	Reaction
G6p_F6p	Upper glycolysis	g6p[c] ⇌ f6p[c]
Pep_Pyr	Lower glycolysis	pep[c] + adp[c] → pyr[c] + atp[c]
G6p_6pgl	PPP	g6p[c] + nadp[c] ⇌ 6pgl[c] + nadph[c]
Pyr_Acald	Ethanol synthesis	pyr[c] → acald[c] + CO_2_[c]
Acald_Eth	Ethanol synthesis	acald[c] + nadh[c] → etoh[c] + nad[c]
Acald_Ac	Acetate metabolism	acald[c] + nadp[c] → ac[c] + nadph[c]
Ac_Accoa	Ac-CoA metabolism	ac[c] + 2 atp[c] → accoa[c] + 2 adp[c]
Pyr_Oaa	TCA reductive branch	pyr[c] + atp[c] + CO_2_[c] → oaa[c] + adp[c]
Acald_Eth_m	Ethanol synthesis	acald[m] + nadh[m] ⇌ etoh[m] + nad[m]
Oaa_Cit_m	TCA oxidative branch	accoa[m] + oaa[m] → cit[m]
Icit_Akg_m_nad	TCA oxidative branch	icit[m] + nad[m] → akg [m] + CO_2_[m] + nadh[m]
Icit_Akg_m_nadp	TCA oxidative branch	icit[m] + nadp[m] → akg[m] + CO_2_[m] + nadph[m]
Eth_t	Ethanol excretion	etoh[c] →
Ac_t	Acetate excretion	ac[c] →
Pyr_t	Pyruvate excretion	pyr[c] →
Akg_t	AKG excretion	akg[c] →
Succ_t	Succinate synthesis	succ[c] →
Glyc_t	Glycerol synthesis	glyc[c] →
CO_2__t	CO_2_ synthesis	CO_2_[c] ⇌
BIOMASS	Biomass formation	3.96 g6p[c] + 0.258 r5p[c] + 0.129 e4p[c] + 0.116 g3p[c] + 0.303 3 pg[c] + 0.232 pep[c] + 0.775 oaa[c] + 1.084 pyr[m] + 0 pyr[c] + 0.176 accoa[m] + 0.252 accoa[c] + 0.106 akg[m] + 0.366 akg[c] + 0 CO_2_[c] + 0.136 glu[c] + 115 atp[c] + 0.106 atp[m] + 1.499 nad[c] + 0.176 nad[m] + 0.602 nadph[m] + 5.35 nadph[c] → 115 adp[c] + 0.106 adp[m] + 1.499 nadh[c] + 0.176 nadh[m] + 0.602 nadp[m] + 5.35 nadp[c]

*flux abbreviations are encoded as substrate and product connected with “_”. For mitochondrial reactions, we added “_m”. Extracellular transport and mitochondrial transport reactions are marked with “_t” and “_tm”, respectively. Metabolite abbreviations can be found in Supplementary Table S2.

For the production of extracellular main metabolites, the corresponding estimated excretion fluxes were chosen as representative. For main metabolic pathways, either the first flux (PPP, upper glycolysis, ethanol synthesis, TCA reductive, TCA oxidative) or the last flux of the pathway (lower glycolysis) were chosen. In the case of parallel fluxes leading to the same metabolite (ethanol synthesis, AKG synthesis), both fluxes were chosen for evaluation.

**Figure 1:**
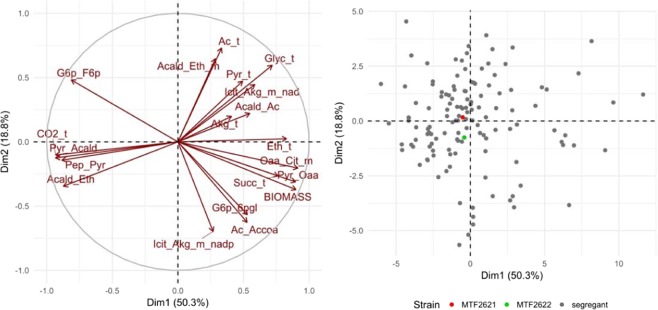
Principle component analysis. PCA of selected estimated fluxes (left), and variation among parents (red and green) and segregant strains (grey) (right). Flux abbreviations are given in Supplementary Tables S2 and S3.

Principal component analysis (PCA) of selected flux reactions was performed to assess flux correlations and to evaluate the variation between parent and segregant strains (Fig. 1).

With the first two dimensions explaining 69.1% of trait variation, the PCA of estimated fluxes adequately depicts the variation among strains. The parent strains behave similarly and show only minor differences in their flux profiles, whereas the segregant strains are more divergent. This finding is confirmed by the visualization of trait distributions (Supplementary Fig. S2). The parental strains are located within the population of segregants for the majority of traits.

**Figure 2:**
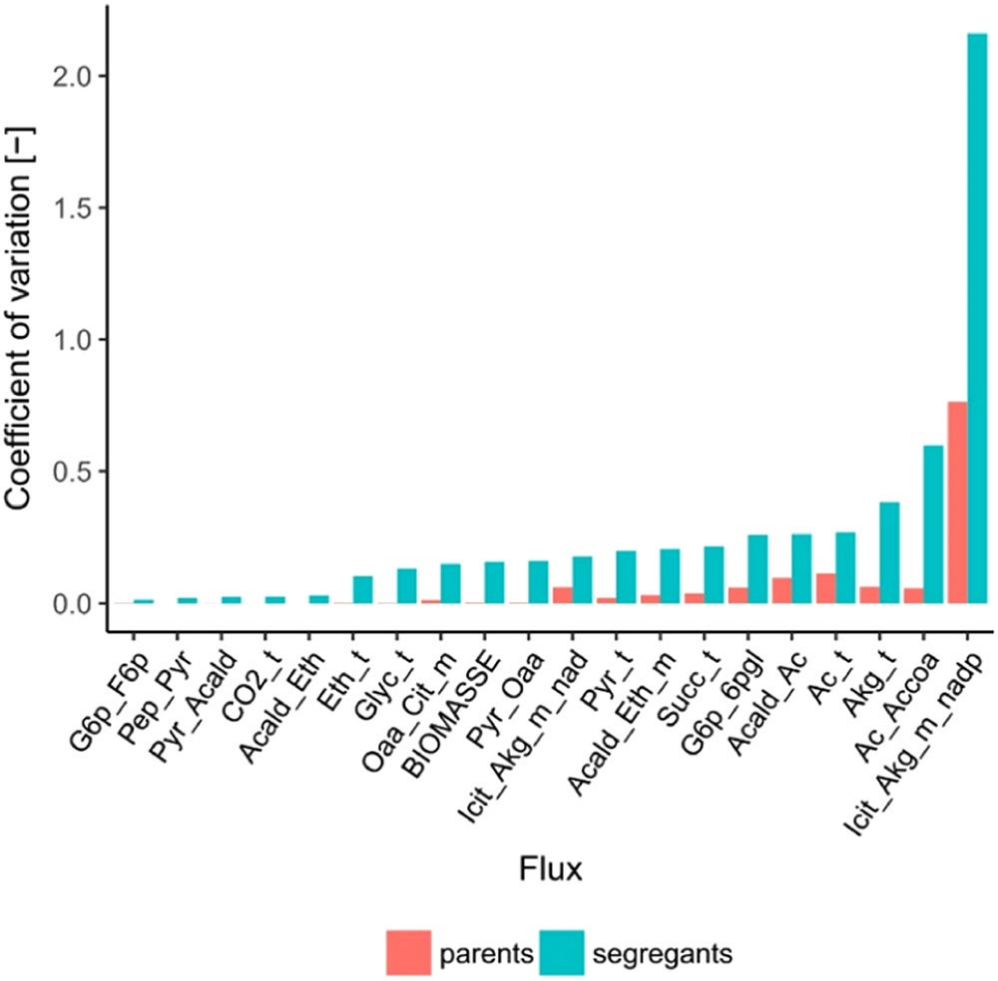
: Flux variation. Coefficient of variation of selected representative fluxes among the parent strains of the study (red) and among the resulting F2-segregants (blue). Flux abbreviations are given in Supplementary Tables S2 and S3.

To further assess variation among strains, coefficients of variation for estimated fluxes were separately calculated for the parent and segregant strains (Fig. 2).

**Figure 3:**
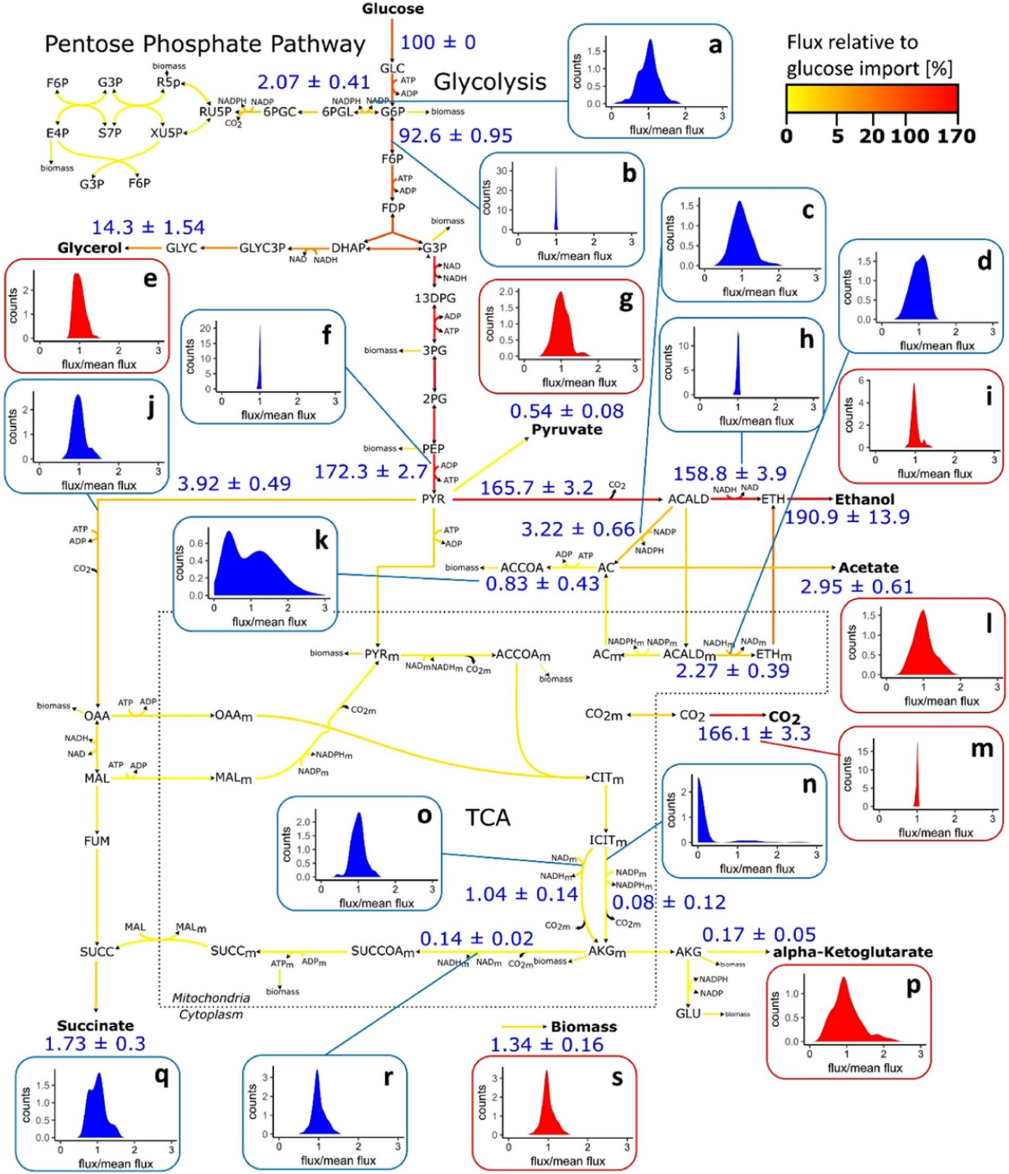
Average flux distribution. Schematic representation of modelled metabolic network with average flux distribution among segregant strains. Flux strength is expressed as percentage of glucose input and is displayed with a colour gradient from yellow to red. Average flux values ± standard deviation are indicated in blue for selected representative metabolic reactions, together with the variation of these fluxes around the mean that is normalized to a value of 1 (**a-s**). Distributions are displayed in red for reactions constrained by experimental data and in blue for modelled reactions. Metabolite abbreviations are given in Supplementary Table S2.

The results demonstrate that the variation among segregant strains regarding CCM fluxes exceeds the variation found between the parent strains, for half of the traits more than ten times (Pep_Pyr, Pyr_Acald, Acald_Eth, Ac_Accoa, Pyr_Oaa, Akg_Succoa_m, Eth_t, Glyc_t, CO2_t, BIOMASSE). This finding confirms the conclusion drawn by PCA (Fig. 1), which is explicitly that the parent strains show higher similarities in flux distributions than the segregant population. However, differences in variation can be seen between single fluxes. To better visualize trait variation between all segregants, the distributions of fluxes were plotted around the mean value for representative reactions within the central carbon metabolic network (Fig. 3).

While the fluxes of glycolysis and ethanol synthesis vary by only ±2.5% around the mean value, the fluxes of the PPP or metabolite production, such as glycerol or acetic acid production, diverge up to 200% around the mean. Most fluxes are normally distributed; however, few outliers can be seen, particularly for *α*-ketoglutarate, pyruvate and ethanol excretion. The distributions of two fluxes differ from a normal distribution, namely, the synthesis of acetyl-CoA from acetate (Ac_Accoa) and the NADP-dependent mitochondrial flux from isocitrate to AKG (Icit_Akg_m_nadp) of the TCA cycle oxidative branch. In the case of Icit_Akg_m_nadp, the analyses indicate that this flux is inactive in the majority of segregant strains and both parents (Supplementary Fig. S2). In the case of Ac_Accoa, a subpopulation of segregants shows a reduced flux towards acetyl-CoA. The presence of two distinctive populations indicates the major influence of one allele on the trait.

### Genome-wide identification of QTLs influencing metabolic carbon fluxes

In the first step, QTL mapping was performed on metabolite yields determined during the exponential phase, using a previously obtained segregant marker map^46^. Linkage analysis led to the detection of 8 QTLs on 5 chromosomes influencing 7 traits (Table 2), which included most of the determined metabolite production yields as well as differences in sugar uptake (expressed as G/F ratio, the ratio of glucose and fructose remaining in the medium).

**Table 2:** Detected metabolite QTLs. List of QTLs with an influence on metabolite yields and the ratio of residual glucose to fructose concentrations (G/F ratio) during the exponential phase.

Trait	QTL name	Chromosome	QTL start [bp]	QTL end [bp]	LOD
Succinate yield	chr4@122.8	IV	356071	380035	4.42
G/F ratio	chr4@125.4	IV	356071	400864	3.67
G/F ratio	chr4@139.3	IV	409433	448045	3.63
CO_2_ yield	chr4@139.3	IV	410742	448045	4.05
Succinate yield	chr4@152.6	IV	426649	488205	4.71
G/F ratio	chr4@160.0	IV	448242	505548	3.76
CO_2_ yield	chr4@160.0	IV	448242	505548	4.33
Glycerol yield	chr7@90.3	VII	252047	273771	3.76
AKG yield	chr10@242.3	X	717987	648141	4.38
Ethanol yield	chr13@208.9	XIII	622064	660267	4.22
G/F ratio	chr13@214.5	XIII	624189	648141	3.62
CO_2_ yield	chr13@214.5	XIII	624189	648141	3.7
Acetate yield	chr13@237.7	XIII	710548	726277	3.84
AKG yield	chr15@31.8	XV	67745	111309	3.52

The highest detected LOD score was 4.71 for QTL chr4@152.6 influencing succinate yield, meaning that almost 16% of trait variation can be explained by this locus.

In the second step, QTL mapping was performed on estimated intracellular carbon fluxes. The analysis detected 24 single results of genomic regions influencing intracellular fluxes (Supplementary Table S4). Loci with highly identical borders were grouped to fQTLs (Table 3).

**Table 3:** Detected fQTLs. List of QTLs with an influence on modelled metabolic fluxes.

Trait	QTL name	Chromosome	QTL start [bp]	QTL end [bp]	LOD max
Glycerol synthesis	chr2@222.9	II	662795	701771	4.58
Malate transport	chr5@128.3	V	354177	400836	4.01
Glycolysis & ethanol synthesis	chr7@18.0	VII	40689	58851	4.63
Biomass	chr7@25.5	VII	52412	82449	3.73
TCA cycle oxidative branch	chr7@25.5	VII	52412	82449	4.05
Transport & excretion TCA metabolites	chr7@25.5	VII	52412	82449	4.05
Ethanol transport	chr8@155.7	VIII	443664	483121	3.45

A total of 4 fQTLs on chromosomes II, V, VII and VIII were detected that influence 7 traits, which includes fluxes of glycolysis/ethanol synthesis, glycerol synthesis, the TCA cycle oxidative branch, biomass formation and metabolite transport/excretion. No QTLs could be detected for the fluxes of the PPP, the TCA cycle reductive branch and glutamate cycle, although these fluxes show the most substantial variation among the segregant strains (Fig. 2). As no QTL with an influence on the synthesis of acetyl-CoA from acetate (Ac_Accoa) was detected, the previous hypothesis from phenotyping that one locus has a major impact on this flux could not be confirmed (Fig. 3). The highest LOD score of 4.63 was found for the influence of QTL chr7@18.0 on glycolysis and ethanol synthesis, meaning that 15.7% of trait variation can be explained by this locus. The QTL region furthermore influences the most traits. In addition to the fluxes of glycolysis and ethanol synthesis, the fluxes of biomass formation, the TCA cycle oxidative branch and metabolite transport/excretion are affected.

**Figure 4:**
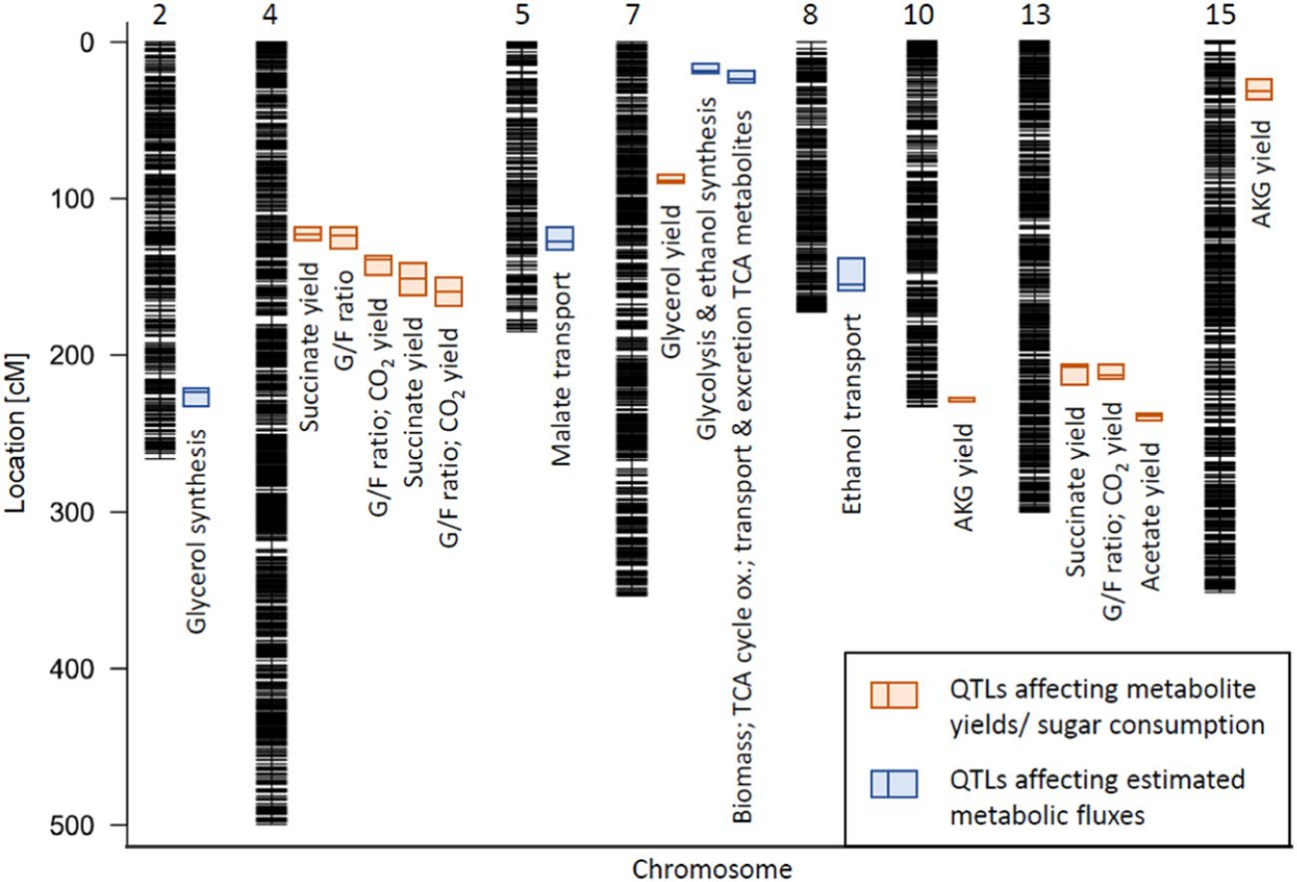
QTL map. Plot of all detected QTLs against related chromosomes with marker positions. QTLs detected to influence metabolite yields or sugar consumption during the exponential phase (orange) and QTLs detected to influence estimated metabolic fluxes (blue) are displayed as 1-LOD support interval with the peak position as horizontal bar.

Combining the results obtained with both QTL mappings, 12 QTLs with a minimum peak distance of 10 cM were detected on 8 chromosomes (Fig. 4), of which 8 QTLs influence metabolite yields or the consumption of sugars during the exponential phase of fermentation, and 4 QTLs influence estimated metabolic fluxes of CCM.

### Validation of detected fQTLs

To evaluate the solidity of obtained fQTL mapping results, candidate genes within the 2 fQTLs with the highest LOD score were selected for validation. These genes were chosen according to their biological function related to central carbon metabolism and their distance to the QTL peak (Table 4).

**Table 4:** Verification of fQTLs. Differences caused by selected allelic gene variants regarding the influenced traits were detected by RHA and are given as the ratio of phenotype MTF2621 to phenotype MTF2622. (p-value: * ≤ 0.05, ** ≤ 0.01).

QTL name	Trait	Evaluated genes	Different impact of allele on trait as MTF2621/MTF2622 [factor]
chr2@222.9	glycerol synthesis	*PDB1*	1.05* glycerol synthesis
chr7@25.5	biomass glycolysis & ethanol synthesis TCA metabolite transport & excretion TCA cycle oxidative branch	*HAP2*	no effect
		*VID30*	0.99* glycolysis & ethanol synthesis 0.92* − 0.73** TCA metabolite excretion (0.90* TCA cycle reductive branch)

**Figure 5:**
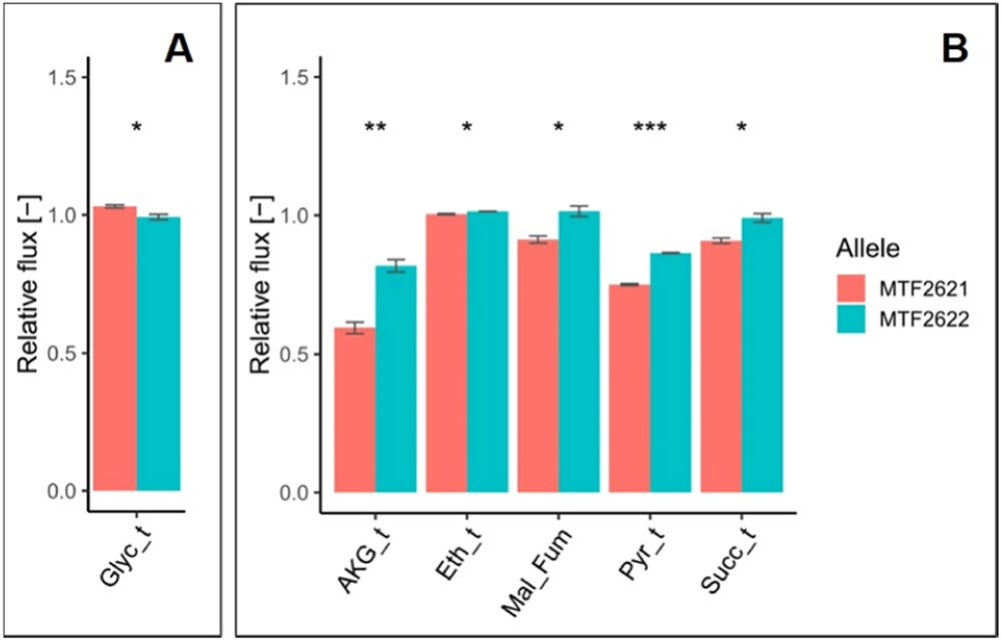
Allelic effect of *PDB1* (**A**) and *VID30* (**B**) on different estimated fluxes of yeast CCM. Fluxes are expressed in relation to the corresponding flux determined in the undeleted heterozygote. (Glyc_t: glycerol excretion; AKG_t: AKG excretion; Eth_t: ethanol excretion; Mal_Fum: malate to fumarate flux; Pyr_t: pyruvate excretion; Succ_t: succinate excretion; p-value: * ≤ 0.05, ** ≤ 0.01, *** ≤ 0.001).

The impact of these genes and their allelic variants was evaluated by RHA. In QTL chr2@222.9, detected to influence glycerol synthesis, *PDB1* with a distance of 22.5 kb to the QTL peak was assessed, and a significant influence of the allelic variants on the trait was detected. The allelic variants of the gene differ in three non-synonymous single-nucleotide polymorphisms (SNPs; Table 5). The MTF2621 allele of *PDB1* increases glycerol synthesis fluxes by 5% (Fig. 5).

**Table 5:** Polymorphisms between target gene alleles. Differences in amino acid (AA) sequences of expressed validated gene variants caused by non-synonymous SNPs between the parent strains. Comparison of the strains MTF2621 and MTF2622 to *S. cerevisiae* type strain S288C.

Gene	Length in AA	AA position	S288C	MTF2621	MTF2622
*PDB1*	366	14	A	A	T
		26	—	A	—
		289	V	V	I
*VID30*	958	37	H	Y	H
		672	E	E	G
		882	I	V	I

In region chrVII:40,689..82,449 detected to influence glycolysis, ethanol synthesis, biomass formation, TCA cycle fluxes and transport/excretion of TCA cycle metabolites, two genes were selected for validation, *HAP2* with a distance of 23.0 kb to the QTL peak, and *VID30* with a distance of 4.0 kb to the QTL peak. While the variants of *HAP2* did not show significant differences regarding estimated metabolic fluxes, the contribution of *VID30* to the detected phenotype variations could be validated (Table 4). The allelic variants of *VID30* differ in three non-synonymous SNPs (Table 5). One SNP lies in the 1000-bp upstream region of the gene at position −318; however, no predicted transcription factor binding site is affected. No SNP was detected in the terminator region. The MTF2621 allele of Vid30 was found to decrease the fluxes of glycolysis and ethanol synthesis in the parental hemizygote by 1% (Fig. 5). The total variation regarding ethanol synthesis is only 6% among the segregant strains (Fig. 3). Although differences between the hemizygotes can not be directly compared to variation between the haploid segregant cells, the determined decrease by 1% caused by the alleles of Vid30 indicates a considerable contribution to total variation. To further evaluate the significance of the determined effect of Vid30 on glycolysis and ethanol synthesis, the accordance of modelled and experimental determined sugar uptake was assessed for all evaluated candidate genes (Supplementary Fig. S3). The mean deviation of the divergence between modelled and measured sugar uptake was 1.2% for triplicate measurements. Therefore, the meaningfulness of the 1% difference in glycolysis/ethanol synthesis found significant for the alleles of VID30 has to be questioned.

In addition to ethanol synthesis, the MTF2621 allele of Vid30 was found to decrease the excretion of pyruvate, *α*-ketoglutarate and succinate by up to 27%. A significant influence of the alleles on the fluxes of the TCA cycle oxidative branch could not be detected. However, the fluxes of the reductive branch of the TCA cycle are significantly affected, with the MTF2621 allele leading to a decrease in fluxes from malate to succinate by 10%.

### Variation of *PDB1* and *VID30* alleles among the *S. cerevisiae* population

**Figure 6:**
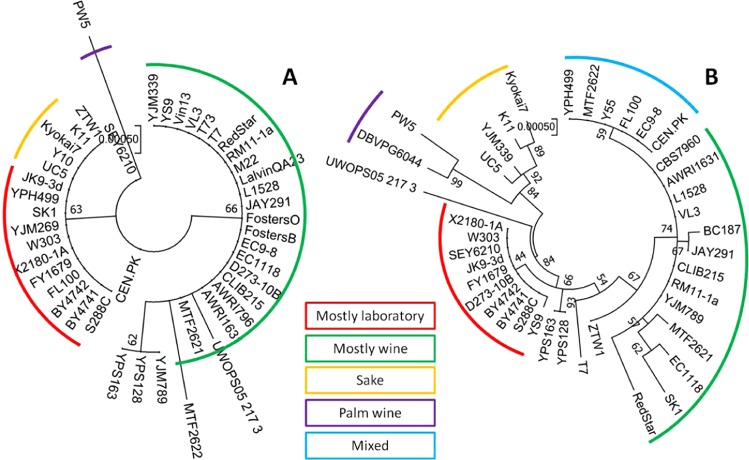
Phylogenetic analysis of target genes. Phylogenetic trees of target genes *PDB1* (**A**) and *VID30* (**B**) made from variant sequences of strains with different origins. Gene sequences were obtained from the *Saccharomyces* Genome Database (https://www.yeastgenome.org). Maximum likelihood trees were constructed by the bootstrap method with 200 replications using MEGA v7.0.26^83^.

To visualize the natural variation of validated target genes within the *S. cerevisiae* population and to potentially link the variants to strain origins, phylogenetic trees were drawn using publicly available *PDB1* and *VID30* gene sequences (Fig. 6 and Supplementary Table S5).

Regarding *PDB1*, allelic variants from yeast strains of different origins do not show much nucleotide variation. Two main clusters can be seen, one consisting of mostly laboratory strains and the other consisting of mostly wine strains. The allelic variants of the parental strains in this study are comparatively close. In contrast, the phylogenetic tree of *VID30* variants displays more variation between strains of different origins. A probable explanation for the larger variation is a larger gene size. Several clusters can be distinguished, including a laboratory strain cluster, a cluster consisting of African and sake strains and a wine strain cluster with a sub cluster of mixed strains. The parental variants are more separated. While the MTF2621 allele of *VID30* is similar to the allele of strain EC1118, a genotypically close wine x flor strain, the MTF2622 allele is located within the mixed cluster.

## Discussion

Many *S. cerevisiae* traits of interest for applications in traditional food industries or industrial biotechnologies are dependent on the distribution of carbon fluxes within yeast CCM. We recently showed the pathway-dependent variability of flux distributions between *S. cerevisiae* strains, which was linked to the strain origin for some fluxes^32^. These findings suggest the existence of a stock of genetic resources that can help us understand the genetic basis of flux distributions and identify relevant targets for yeast strain improvement. In recent years, powerful methods, such as QTL mapping, have been developed to link phenotypic and genomic variations. Our objective was to assess the potential of QTL mapping to detect genomic regions influencing metabolic fluxes (fQTLs).

To this end, we used a population of 130 F2-segregants obtained from a cross of two wine yeast strains. For these strains, intracellular carbon fluxes were estimated by applying concentrations of extracellular metabolites during the exponential growth phase to a constraint-based stoichiometric model of yeast CCM. Analysis of flux deviations within the population of segregants indicated a positive correlation between the fluxes of the PPP, the TCA cycle oxidative branch and biomass formation, whereas these fluxes were negatively correlated to the fluxes of upper glycolysis. Negative correlations were furthermore found between the fluxes of lower glycolysis/ethanol synthesis and glycerol formation (Fig. 1). These observations are consistent with previous studies^32,50^.

Although the parent strains did not display large variation for most modelled fluxes, substantial variation among the segregants was observed (Fig. 2). For some fluxes, for example, the PPP or main metabolic synthesis fluxes, the variation among segregants reached the variation among *S. cerevisiae* strains from different ecological origins as determined by Nidelet *et al*. 2016^32^. This finding emphasizes the complex nature of intracellular flux distributions and indicates a rich genomic resource for metabolic profile optimization.

With 8 detected QTLs, the number of regions influencing metabolite yields during the exponential phase was higher than the detected 4 QTLs influencing modelled metabolic fluxes. However, the regions from both linkage analyses differ from each other (Fig. 4), demonstrating that the modelling step was crucial for fQTL detection. While QTL mapping of metabolite yields naturally considers a single information, the modelling step preceding fQTL mapping represents the integration of multiple information.

As reported in the literature and seen in our study, variances of certain intracellular fluxes, such as glycolysis, are generally low. As result, increased statistical power is needed for a more thorough determination of the impact of genomic variation on these fluxes. This determination could be achieved by increasing the number of segregants or by performing strategies of multiple QTL mapping, which has the potential to find QTLs with minor contributions.

All QTLs were compared to 8 loci detected during our previous study to influence extracellular metabolite production yields after 80% of the fermentation using the same parent strains^46^. Only QTL chr7@18.0, which influences the fluxes of glycolysis, ethanol synthesis, biomass production, the TCA cycle and transport/excretion of TCA cycle metabolites, was detected in our previous study to influence pyruvate production yield after 80% of the fermentation. This finding indicates that the impact of QTL chr7@18.0 on flux distributions has a long-lasting effect on metabolite formation that can still be detected at the end of fermentation. However, considering the concentrations of extracellular metabolites during the exponential phase and after 80% of the fermentation, all detected QTL regions differ from each other, and there are no common QTLs affecting metabolite production during both phases of fermentation. This finding indicates that different genomic regions could control metabolite production in the growth phase and stationary phase, which actually corresponds to very different physiologic states of yeast during fermentation.

Assessment of the two detected fQTLs with the highest LOD scores by RHA revealed the implication of *PDB1* and *VID30* in the evaluated traits. *PDB1*, shown to influence the fluxes of glycerol synthesis (Table 4, Fig. 5), encodes the beta subunit of pyruvate dehydrogenase (PDH), which is part of the large multienzyme PDH complex^51^. The PDH complex, which includes the other components dihydrolipoamide acetyltransferase and dihydrolipoamide dehydrogenase, converts pyruvate into acetyl-CoA^52^. The allelic variants of *PDB1* differ in three non-synonymous SNPs (Table 5), one of which, SNP V289I, lies in the pyruvate-ferredoxin oxidoreductase domain II of the protein. A possible explanation for the impact of Pdb1 variants on glycerol fluxes is that the MTF2621 allele of Pdb1 shows an increased conversion rate of pyruvate to acetyl-CoA, which leads to a higher formation of the redox cofactor NADH. The resulting cofactor excess is then compensated through increased glycerol synthesis, which maintains redox balance by NADH consumption^53^. However, no significant difference in the estimated flux from pyruvate to acetyl-CoA (Pyr_Accoa_m) was detected by RHA for the alleles of *PDB1*.

The second validated gene, *VID30*, possibly influences the fluxes of glycolysis/ethanol synthesis and has an impact on the TCA cycle reductive branch and the excretion of TCA cycle metabolites (Table 4, Fig. 5). Two functions of Vid30 could potentially account for these observed differences: the regulation of genes involved in glutamate/glutamine synthesis and the degradation of various metabolic enzymes. The expression of *VID30* is repressed by ammonia and upregulated in response to low ammonia levels, a characteristic limitation during fermentation in grape must, which was the case in this study. Vid30 regulates various nitrogen catabolic genes, including *GDH1*, *GDH2*, *GDH3*, *GLN1* and *GLT1*. These genes express enzymes involved in the synthesis (and interconversion) of glutamate and glutamine from AKG and ammonia, therefore explaining the role of Vid30 in central carbon metabolism since AKG is part of the fluxes of the TCA cycle oxidative branch. Gdh1, Gdh3 and Gln1 catalyse reactions from AKG to glutamine^54–56^, whereas Gdh2 catalyses the conversion of glutamate to AKG^57^. Glt1 synthesizes glutamate from either AKG or glutamine^58^. In low ammonia environments, Vid30 behaves as a positive regulator of *GDH1*, *GDH3* and *GLT1*, which increases the flux from AKG to glutamate^59^. Since decreased AKG production was detected for the MTF2621 allele of Vid30 (Fig. 5), we suggest that this variant stimulates increased flux from AKG to glutamate through the positive regulation of *GDH1*, *GDH3* and *GLT1*.

Another potential role of Vid30 in central carbon metabolism is its regulation of various metabolic enzymes through degradation. When glucose-starved yeast is again transferred to glucose-rich medium, e.g., during inoculation, metabolism increases the expression of glycolytic enzymes and simultaneously inactivates gluconeogenetic enzymes through catabolite inactivation. Vid30 possesses two functions in this process. It acts as a subunit of the glucose induced degradation (GID) protein complex that performs the ubiquitination of enzymes, which leads to their proteasome dependent inactivation^60–63^. Furthermore, Vid30 plays an important role in the formation of vesicles of the vacuole import and degradation pathway^64^, which carries out the degradation of enzymes expressed under the growth on non-fermentable carbon sources^65–67^. Regulation performed in this manner includes the turnover of hexose transporters Hxt3 and Hxt7^68,69^. In addition, various enzymes are regulated through degradation by Vid30 that catalyse gluconeogenesis reactions, such as fructose-1,6-bisphosphatase, cytosolic malate dehydrogenase, isocitrate lyase and phosphoenolpyruvate carboxykinase^70–73^. The reactions catalysed by these enzymes strongly affect the fluxes of glycolysis and the TCA cycle.

The allelic variants of *VID30* differ in three non-synonymous SNPs (Table 5), one of which, SNP V882I, lies in the CTLH/CRA domain of the protein, a protein-protein interaction domain also found in other components of the GID complex. We propose that the SNPs in the MTF2621 variant of Vid30 influence the protein’s ability to inactivate hexose transporters and gluconeogenesis enzymes by degradation, hypothetically by an altered affinity to other components of the GID complex. This hypothesis is supported by the observed influence of allelic variants on the fluxes of the TCA cycle reductive branch (Fig. 5), as cytosolic malate dehydrogenase, which catalyses the reaction from malate to oxaloacetate, is among the enzymes inactivated by Vid30^65^. Furthermore, the reported SNPs could affect the role of Vid30 in the regulation of enzymes involved in the synthesis of glutamate from AKG. This hypothesis is supported by the detected significant influence of Vid30 alleles on AKG formation (Fig. 5). On the other hand, a significant difference between the evaluated alleles in the flux from AKG to glutamate could not be detected by RHA. The difference in AKG formation could also be explained by the role of Vid30 in the degradation of isocitrate lyase. The enzyme catalyses the reaction from isocitrate to succinate, which could influence AKG synthesis.

## Conclusion

In this study, we prove the feasibility of using modelled phenotypic data to detect regions in the genome influencing the distribution of carbon fluxes within central carbon metabolism (fQTLs) in *S. cerevisiae*. We used concentrations of extracellular main metabolites during the exponential phase of yeast fermentation to estimate intracellular fluxes, applying a constraint-based model. This strategy allowed the integration of otherwise independent quantifiable traits and resulted in the detection of 4 fQTLs with an influence on 4 main metabolic pathways and various metabolite transport and excretion fluxes. These QTLs could not have been found by linkage analysis considering extracellular metabolite concentrations alone, demonstrating the need for the modelling step.

The solidity of our approach was further confirmed by the validation of two target genes within the identified fQTLs, *PDB1* and *VID30*. The allelic variants of *PDB1* account for variation in the glycerol synthesis flux, which we hypothesize to be caused by redox imbalances as a result of altered pyruvate conversion. The variants of *VID30* impact the fluxes of glycolysis, ethanol synthesis and the TCA cycle, which we propose to be caused by differences in the regulation of enzymes catalysing glutamate formation or differences in the catabolite-induced degradation of enzymes involved in sugar uptake, gluconeogenesis and the TCA cycle. Compared to strains of other origins, the parental variants of the evaluated target genes are comparatively close. The characterization of more distant variants and an evaluation of their influence on intracellular flux distributions will disclose genetic resources that bear further potential to shape the metabolic profile of strains.

In summary, our findings of fQTLs and allelic variants impacting metabolic fluxes increase our knowledge of the links between genomic variation and yeast metabolic properties and provide a proof-of-concept for the applicability of QTL mapping on modelled metabolic fluxes. This result offers exciting opportunities for uncovering superior allelic variants impacting these traits, which could be used to improve strains for manifold purposes, e.g., the production of biofuels or other bulk and fine chemicals.

## Materials and Methods

### Media

Yeast strains were cultured at 28 °C in yeast extract peptone dextrose (YPD) media, containing 10 g/L yeast extract, 20 g/L peptone and 20 g/L glucose. Solid YPD media contained 1.5% agar. Selective YPD media contained 200 µg/mL geneticin (G418), 200 µg/mL nourseothricin (clonNAT) or 200 µg/mL hygromycin B.

Fermentations were carried out in synthetic grape must (SM200) as described by Bely *et al*. 1990^74^. The medium contains glucose and fructose at a concentration of 100 g/L each, and assimilable nitrogen in the form of ammonium and free amino acids at a concentration of 200 mg/L.

### Strain generation

This study used a set of 130 F2-segregants generated in a former study to map QTLs for main and volatile metabolite production by *S. cerevisiae* during alcoholic fermentation^46^. The parent strains of this segregant population are strain MTF2621 (haploid spore of 4CAR1 [Δ*HO*::*Neo*^r^]) and MTF2622 (haploid spore of T73 [Δ*HO*::*Nat*^r^]). Strain T73 belongs to the phylogenetic clade of wine strains, whereas strain 4CAR1 belongs to the group of champagne strains, which originated through crossings between strains of the wine clade and the flor clade^75^.

### Genotyping of strains

The segregant strain marker map used for linkage analysis in this study was generated during a previous study in our working group^46^. Parent and segregant strains were genotyped by whole genome sequencing using Illumina technology, and a global set of 18155 biallelic variant positions was obtained. This set was reduced to a dataset of 3727 SNP markers with a minimum spacing of 2.0 kb between SNPs. Four segregant strains with the most ambiguous markers were excluded to increase the meaningfulness of the analysis. One strain was excluded because of its close genomic proximity to another segregant. This exclusion left a population of 125 F2-segregants for statistical analyses. Information available in the *Saccharomyces* Genome Database (https://www.yeastgenome.org) was used to associate SNPs with annotated protein domains. The effect of detected SNPs on putative transcription factor binding sites was analysed using YEASTRACT (release 2017)^76^.

### Phenotyping of strains

Segregant strains were phenotyped in duplicate with the parent strains as a control. Sterilized 300-mL glassware mini fermenters were filled with 280 mL of SM200 (synthetic must with 200 mg/L of assimilable nitrogen) and closed with an air lock. The fermenters were inoculated with overnight yeast cultures to a cell density of 1 × 10^6^ cells/mL, weighed and left at 24 °C under stirring (300 rpm). Fermentations were sampled during the exponential phase (when 10 g/L of CO_2_ was produced), which was determined by weighting the fermenters regularly. For each fermentation, dry biomass was determined in duplicate by filtering 10 mL of cell suspension through a nitrocellulose membrane with a porosity of 0.45 μm (Millipore, France) and known dry weight. The membrane was rinsed twice with 10 mL of distilled water, dried for 48 h and weighed to determine the dry biomass of the sample. The concentrations of unfermented sugars (glucose and fructose) and extracellular carbon metabolites (ethanol, glycerol, acetic acid, succinic acid, pyruvic acid, and *α*-ketoglutaric acid) in the fermentation medium were determined by high-performance liquid chromatography (HPLC). The flow rate of the device (HPLC 1290 Infinity, Agilent Technologies, USA) was set to 0.6 mL/min (0.005 N H_2_SO_4_). Samples were separated by a pre-column and an ion-exclusion column (Phenomenex REZEX™ ROA-Organic Acid H +(8%)), which was thermostatically controlled at 60 °C. Compounds were detected using a refractometer in combination with a UV spectrometer at 210 nm. Chromatograms were processed with Agilent EZChrom software.

### Modelling of metabolic fluxes

To predict intracellular metabolic fluxes, we used DynamoYeast, a previously developed model of yeast central carbon metabolism^18^. This model has been chosen for several reasons. First, it had been designed specifically to predict fluxes in oenological fermentation. It covers *S. cerevisie* central carbon metabolism with the specification of anaerobic metabolism, e.g., TCA fluxes are composed of an oxidative and reductive branch and therefore do not form a cycle. Second, the biomass reaction function of the model had been calibrated with wine yeast strain EC1118, which is phylogenetically closer to the parent strains used in this study than the widely used laboratory reference strain^18^. Third, it had been used and validated in two previous projects in very similar conditions^18,32^. Last, the choice of a comparatively small model (favored over a genome-scale model) is consistent with other studies in literature that use large scale models to examine qualitative phenotypes^13^, whereas small-scale models are used for quantitative modelling^20–22,77^.

The DynamoYeast model covers 68 reactions (Supplementary Table S3) and 61 metabolites (Supplementary Table S2) of central carbon metabolism, and distinguishes three compartments, the mitochondria, cytoplasm and extracellular excretion. Extracellular metabolite concentrations (in mmol/L) and dry mass weight (in g/L) obtained by the phenotyping of segregant strains during the middle of the exponential phase were used to constrain the model. The error margin for the metabolite reaction rate boundaries of the model was set to ± 2.5%. Flux distribution throughout the metabolic network was obtained for each segregant by mass balance analysis with a minimization of glucose input as the objective function. As consequence, our method directly computes mass distribution, unlike other constraint-based methods, e.g., metabolic flux analysis (Vallino and Stephanopoulos, 1993^78^), that derive mass data to obtain flux distributions (see Celton *et al*. 2012^18^ for details about the methodology). For computing mass distribution, we follow the assumption that all fluxes - along with the biomass composition - are constant during the exponential phase. The estimation of mass distribution allows for the expression of fluxes as yields instead of reaction rates.

Fructose was treated as glucose in the modelling approach as this did not impact flux predictions. Finally, estimated fluxes were normalized to the predicted sugar uptake in order to enable the comparison of relative flux distributions between strains. Due to block effects concerning the determination of succinate concentrations, succinate excretion fluxes were constrained by setting a fixed range that corresponded to the maximum succinate excretion flux variation between *S. cerevisiae* strains determined in a previous study by our working group^32^.

All predictions were performed using the programming language R v3.2.3 with the R/sybil v2.0.0 and R/sybilSBML v2.0.11 libraries^79^.

### QTL mapping

QTL mapping of modelled metabolic fluxes was carried out by single QTL mapping (interval mapping method) using the genotype and phenotype information of segregant strains. Linkage analysis was performed using the programming language R v3.2.3 (https://www.r-project.org) with the R/qtl v1.40–8 and R/eqtl v1.1–7 libraries^80^. Two different phenotype models were tested, the normal model using Haley-Knott regression and non-parametric analysis. The statistical analyses resulted in logarithm of odds (LOD) scores for each marker and pseudo-marker every 2.5 cM. Individual LOD score thresholds for a false discovery rate of 0.05 were determined with 1000 permutations. If the same locus was detected with both phenotype models, the results with the higher LOD score were selected. An interval estimate of the location of each QTL was obtained as the region in which the LOD score is within 1 unit of the peak LOD score (1-LOD support interval). QTL mapping results for single metabolic reactions were considered to be a common fQTL if their peaks were less than 10 cM apart.

### Reciprocal hemizygosity analysis

Molecular dissection of the detected QTLs was performed by RHA^81,82^. Target genes in QTLs were chosen according to a biological function associated with central carbon metabolism and the gene’s proximity to the determined QTL peak. The gene sequences were deleted in both parent strains by homologous recombination with a disruption cassette containing the hygromycin B resistance gene (*hph*^r^). The disruption cassettes were amplified by polymerase chain reaction (PCR) of the plasmid pAG32 with the primers del_(GENE)_fw and del_(GENE)_rv (Supplementary Table S1). Positive integration was selected by plating the transformed cells on YPD-agar plates containing hygromycin B. Correct gene deletion was verified by PCR using primer test_(GENE)_fw, which binds upstream of the deleted gene, and primer Hygro_rv, which binds within the deletion cassette. Subsequently, deleted parent strains were mated with the opposite undeleted parent to form a heterozygote that is hemizygous for the target gene. Hemizygous constructions were phenotyped in triplicate. The significance of the influence of allelic gene variants on modelled metabolic fluxes was evaluated by student’s t-test. If a variant’s impact on several fluxes was tested, p-values were not adjusted for multiple comparisons.

## Supplementary information

Supplementary Information.

## Data Availability

Genome sequencing data generated during the current study is available from NCBI under bioproject number PRJNA433287; SNP data, marker map and phenotypic data set are available from the INRA Dataverse: https://data.inra.fr/dataset.xhtml?persistentId = doi:10.15454/C1F8MO.
