# Supplementary material for: QTL mapping of modelled metabolic fluxes reveals gene variants impacting yeast central carbon metabolism
Source: Sci Rep. 2020 Feb 7;10:2162. doi: 10.1038/s41598-020-57857-3 (PMC7005809; doi:10.1038/s41598-020-57857-3)
Supplement: Supplementary file 1 — Supplementary Information. [file 41598_2020_57857_MOESM1_ESM.pdf]

QTL mapping of modelled metabolic fluxes reveals gene variants impacting yeast central carbon metabolism

Matthias Eder[a], Thibault Nidelet[a], Isabelle Sanchez[a,b], Carole Camarasa[a], Jean-Luc Legras[a], Sylvie Dequin[a,*]

[a]SPO, Univ Montpellier, INRA, Montpellier SupAgro, F-34060 Montpellier, France

[b]MISTEA, INRA, Montpellier SupAgro, F-34060 Montpellier, France

*Corresponding author. Mailing address: INRA, 2 Place Pierre Viala, 34060 Montpellier CEDEX 2, France; Phone: +33 4 99 61 25 28; E-mail: sylvie.dequin@inra.fr

Supplementary Information

Supplementary Figure S1: Correlation of the null space of the DynamoYeast model. Each square represents the correlation value between two reactions of the model (from red -1 to dark blue +1). The correlation matrix was obtained by sampling of the null space of the model following the method of Poolman et al. (2007)[49].

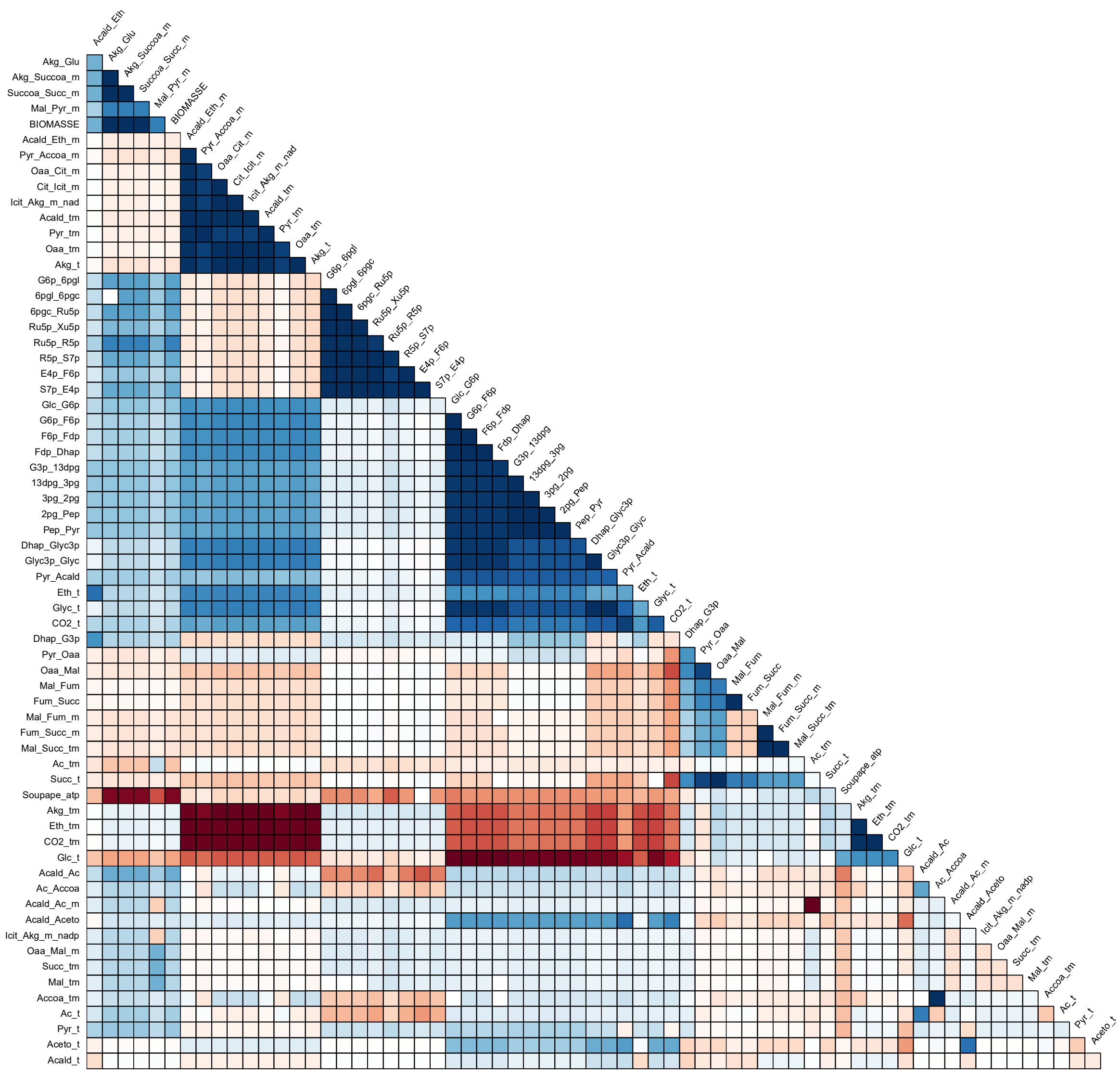

Supplementary Table S1: List of primers used in this study with nucleotide sequences (5' -> 3')

Primer name	Nucleotide sequence (5' -> 3')
del_HAP2_fw	TGGAAGAGGAACAAGAACGCCATGTCAGCAGACGAAACGGTTCGTACGCTGCAGGTCGAC
del_HAP2_rv	TAAATAGGCCATATGGATACCATGTGGTATAAGAGGGCACGCATAGGCCACTAGTGGATCTG
del_PDB1_fw	CCTGTGTTTGTTCATTGATAATCGATCGCAGTTTAGTAAGTTCGTACGCTGCAGGTCGAC
del_PDB1_rv	ACTATTTCCGCGAAGAGGGTAGAAAGTGTAGGGTACAGGGGCATAGGCCACTAGTGGATCTG
del_VID30_fw	CGTTAAAGCCAAGCGTCGAATTTCAGCATAATTAAGAGGATTCGTACGCTGCAGGTCGAC

del_VID30_rv	ATGACTGATATCACATGGCTTTGTTGTTTGAAGGTGCTTGGCATAGGCCACT AGTGGATCTG
Hygro_rv	TGTTATGCGGCCATTGTC
test_HAP2_fw	CGTACAGCCATTGACCATAG
test_PDB1_fw	AATTCCGCCTCCCTCATAAC
test_VID30_fw	ACCTCTACTTCGACCATCAC

Supplementary Table S2: Abbreviations for the metabolites of CCM and their compartmental localization.

**Metabolite abbreviation**	**Compartment**	**Metabolite description**
13dpg[c]	cytoplasm	3-Phospho-D-glyceroyl-phosphate
2pg[c]	cytoplasm	D-Glycerate-2-phosphate
3pg[c]	cytoplasm	3-Phospho-D-glycerate
6pgc[c]	cytoplasm	6-Phospho-D-gluconate
6pgl[c]	cytoplasm	6-phospho-D-glucono-1,5-lactone
ac[c]	cytoplasm	Acetate
ac[m]	mitochondria	Acetate
acald[c]	cytoplasm	Acetaldehyde
acald[m]	mitochondria	Acetaldehyde
accoa[c]	cytoplasm	Acetyl-CoA
accoa[m]	mitochondria	Acetyl-CoA
adp[c]	cytoplasm	ADP
adp[m]	mitochondria	ADP
akg[c]	cytoplasm	Alpha ketoglutarate
akg[m]	mitochondria	Alpha ketoglutarate
amp[m]	mitochondria	AMP
atp[c]	cytoplasm	ATP
atp[m]	mitochondria	ATP
cit[m]	mitochondria	Citrate
CO_2[c]	cytoplasm	CO_2
CO_2[m]	mitochondria	CO_2
coa[c]	cytoplasm	Coenzyme-A
coa[m]	mitochondria	Coenzyme-A
dhap[c]	cytoplasm	Dihydroxyacetone-phosphate
e4p[c]	cytoplasm	D-Erythrose-4-phosphate
etoh[c]	cytoplasm	Ethanol

etoh[m]	mitochondria	Ethanol
f6p[c]	cytoplasm	D-Fructose-6-phosphate
fdp[c]	cytoplasm	D-Fructose-1,6-bisphosphate
fum[c]	cytoplasm	Fumarate
fum[m]	mitochondria	Fumarate
g3p[c]	cytoplasm	Glyceraldehyde-3-phosphate
g6p[c]	cytoplasm	D-Glucose-6-phosphate
glc[c]	cytoplasm	D-Glucose
gln[c]	cytoplasm	L-Glutamine
glu[c]	cytoplasm	L-Glutamate
glu[m]	mitochondria	L-Glutamate
glyc[c]	cytoplasm	Glycerol
glyc3p[c]	cytoplasm	Glycerol-3-phosphate
icit[m]	mitochondria	Isocitrate
mal[c]	cytoplasm	L-Malate
mal[m]	mitochondria	L-Malate
nad[c]	cytoplasm	Nicotinamide-adenine-dinucleotide
nad[m]	mitochondria	Nicotinamide-adenine-dinucleotide
nadh[c]	cytoplasm	Nicotinamide-adenine-dinucleotide-reduced
nadh[m]	mitochondria	Nicotinamide-adenine-dinucleotide-reduced
nadp[c]	cytoplasm	Nicotinamide-adenine-dinucleotide-phosphate
nadp[m]	mitochondria	Nicotinamide-adenine-dinucleotide-phosphate
nadph[c]	cytoplasm	Nicotinamide-adenine-dinucleotide-phosphate-reduced
nadph[m]	mitochondria	Nicotinamide-adenine-dinucleotide-phosphate-reduced
oaa[c]	cytoplasm	Oxaloacetate
oaa[m]	mitochondria	Oxaloacetate
pyr[c]	cytoplasm	Pyruvate
pyr[m]	mitochondria	Pyruvate
r5p[c]	cytoplasm	alpha-D-Ribose-5-phosphate
ru5p[c]	cytoplasm	D-Ribulose-5-phosphate
s7p[c]	cytoplasm	Sedoheptulose-7-phosphate
succ[c]	cytoplasm	Succinate

succ[m]	mitochondria	Succinate
succoa[m]	mitochondria	Succinyl-CoA
xu5p[c]	cytoplasm	D-Xylulose-5-phosphate

Supplementary Table S3: Estimated metabolic fluxes with their corresponding compartment.

Flux abbreviation*	Compartment	Reaction
Glc_G6p	cytoplasm	glc[c] + atp[c] → g6p[c] + adp[c]
G6p_F6p	cytoplasm	g6p[c] ⇌ f6p[c]
F6p_Fdp	cytoplasm	f6p[c] + atp[c] → fdp[c] + adp[c]
Fdp_Dhap	cytoplasm	fdp[c] ⇌ dhap[c] + g3p[c]
Dhap_G3p	cytoplasm	dhap[c] ⇌ g3p[c]
G3p_13dpg	cytoplasm	g3p[c] + nad[c] ⇌ 13dpg[c] + nadh[c]
13dpg_3pg	cytoplasm	13dpg[c] + adp[c] ⇌ 3pg[c] + atp[c]
3pg_2pg	cytoplasm	3pg[c] ⇌ 2pg[c]
2pg_Pep	cytoplasm	2pg[c] ⇌ pep[c]
Pep_Pyr	cytoplasm	pep[c] + adp[c] → pyr[c] + atp[c]
G6p_6pgl	cytoplasm	g6p[c] + nadp[c] ⇌ 6pgl[c] + nadph[c]
6pgl_6pgc	cytoplasm	6pgl[c] → 6pgc[c]
6pgc_Ru5p	cytoplasm	6pgc[c] + nadp[c] → CO_2[c] + nadph[c] + ru5p[c]
Ru5p_Xu5p	cytoplasm	ru5p[c] ⇌ xu5p[c]
Ru5p_R5p	cytoplasm	ru5p[c] ⇌ r5p[c]
R5p_S7p	cytoplasm	r5p[c] + xu5p[c] ⇌ g3p[c] + s7p[c]
E4p_F6p	cytoplasm	e4p[c] + xu5p[c] ⇌ f6p[c] + g3p[c]
S7p_E4p	cytoplasm	g3p[c] + s7p[c] ⇌ e4p[c] + f6p[c]
Dhap_Glyc3p	cytoplasm	dhap[c] + nadh[c] → glyc3p[c] + nad[c]
Glyc3p_Glyc	cytoplasm	glyc3p[c] → glyc[c]
Pyr_Acald	cytoplasm	pyr[c] → acald[c] + CO_2[c]
Acald_Eth	cytoplasm	acald[c] + nadh[c] → etoh[c] + nad[c]
Acald_Ac	cytoplasm	acald[c] + nadp[c] → ac[c] + nadph[c]
Ac_Accoa	cytoplasm	ac[c] + 2 atp[c] → accoa[c] + 2 adp[c]
Pyr_Oaa	cytoplasm	pyr[c] + atp[c] + CO_2[c] → oaa[c] + adp[c]
Acald_Eth_m	mitochondria	acald[m] + nadh[m] ⇌ etoh[m] + nad[m]
Acald_Ac_m	mitochondria	acald[m] + nadp[m] → ac[m] + nadph[m]

Oaa_Mal	cytoplasm	oaa[c] + nadh[c] ⇌ mal[c] + nad[c]
Mal_Fum	cytoplasm	mal[c] → fum[c]
Fum_Succ	cytoplasm	fum[c] → succ[c]
Akg_Glu	cytoplasm	akg[c] + nadph[c] ⇌ glu[c] + nadp[c]
Glu_Akg_m	mitochondria	glu[m] + nad[m] → akg[m] + nadh[m]
Pyr_Accoa_m	mitochondria	pyr[m] + nad[m] → accoa[m] + nadh[m] + CO_2[m]
Oaa_Cit_m	mitochondria	accoa[m] + oaa[m] → cit[m]
Cit_Icit_m	mitochondria	cit[m] ⇌ icit[m]
Icit_Akg_m_nad	mitochondria	icit[m] + nad[m] → akg [m]+ CO_2[m] + nadh[m]
Icit_Akg_m_nadp	mitochondria	icit[m] + nadp[m] → akg[m] + CO_2[m] + nadph[m]
Akg_Succoa_m	mitochondria	akg[m] + nad[m] → succoa[m] + CO_2[m] + nadh[m]
Succoa_Succ_m	mitochondria	succoa[m] + adp[m] → succ[m] + atp[m]
Oaa_Mal_m	mitochondria	oaa[m] + nadh[m] → mal[m] + nad[m]
Mal_Fum_m	mitochondria	mal[m] → fum[m]
Fum_Succ_m	mitochondria	fum[m] → succ[m]
Mal_Pyr_m	mitochondria	mal[m] + nadp[m] → CO_2[m] + nadph[m] + pyr[m]
Acald_tm	transport	acald[c] ⇌ acald[m]
Succ_tm	transport	succ[c] + atp[c] → succ[m] + adp[c]
Mal_tm	transport	mal[c] + atp[c] → mal[m] + adp[c]
Mal_Succ_tm	transport	mal[c] + succ[m] ⇌ mal[m] + succ[c]
Pyr_tm	transport	pyr[c] + atp[c] → pyr[m] + adp[c]
Akg_tm	transport	akg[c] ⇌ akg[m]
Oaa_tm	transport	oaa[c] + atp[c] → oaa[m] + adp[c]
Eth_tm	transport	etoh[c] ⇌ etoh[m]
CO_2_tm	transport	CO_2[c] ⇌ CO_2[m]
Ac_tm	transport	ac[c] ⇌ ac[m]
Accoa_tm	transport	accoa[c] → accoa[m]
Glu_tm	transport	glu[c] + atp[c] → glu[m] + adp[c]
Glc_t	transport	glc[c] →
Eth_t	transport	etoh[c] →
Ac_t	transport	ac[c] →
Pyr_t	transport	pyr[c] →
Akg_t	transport	akg[c] →
Succ_t	transport	succ[c] →

But_t	transport	but[c] →
Aceto_t	transport	aceto[c] →
Acald_t	transport	acald[c] →
Glyc_t	transport	glyc[c] →
CO_2_t	transport	CO_2[c] ⇌
ATP_Shuttle	cytoplasm	atp ⇌ adp
BIOMASS	biomass	3.96 g6p[c] + 0.258 r5p[c] + 0.129 e4p[c] + 0.116 g3p[c] + 0.303 3pg[c] + 0.232 pep[c] + 0.775 oaa[c] + 1.084 pyr[m] + 0 pyr[c] + 0.176 accoa[m] + 0.252 accoa[c] + 0.106 akg[m] + 0.366 akg[c] + 0 CO_2[c] + 0.136 glu[c] + 115 atp[c] + 0.106 atp[m] + 1.499 nad[c] + 0.176 nad[m] + 0.602 nadph[m] + 5.35 nadph[c] → 115 adp[c] + 0.106 adp[m] + 1.499 nadh[c] + 0.176 nadh[m] + 0.602 nadp[m] + 5.35 nadp[c]

* flux abbreviations are encoded as substrate and product connected with "_". For mitochondrial reactions, we added "_m". Extracellular transport and mitochondrial transport reactions are marked with "_t" and a "_tm", respectively. Metabolite abbreviations can be found in Supplementary Table S2.

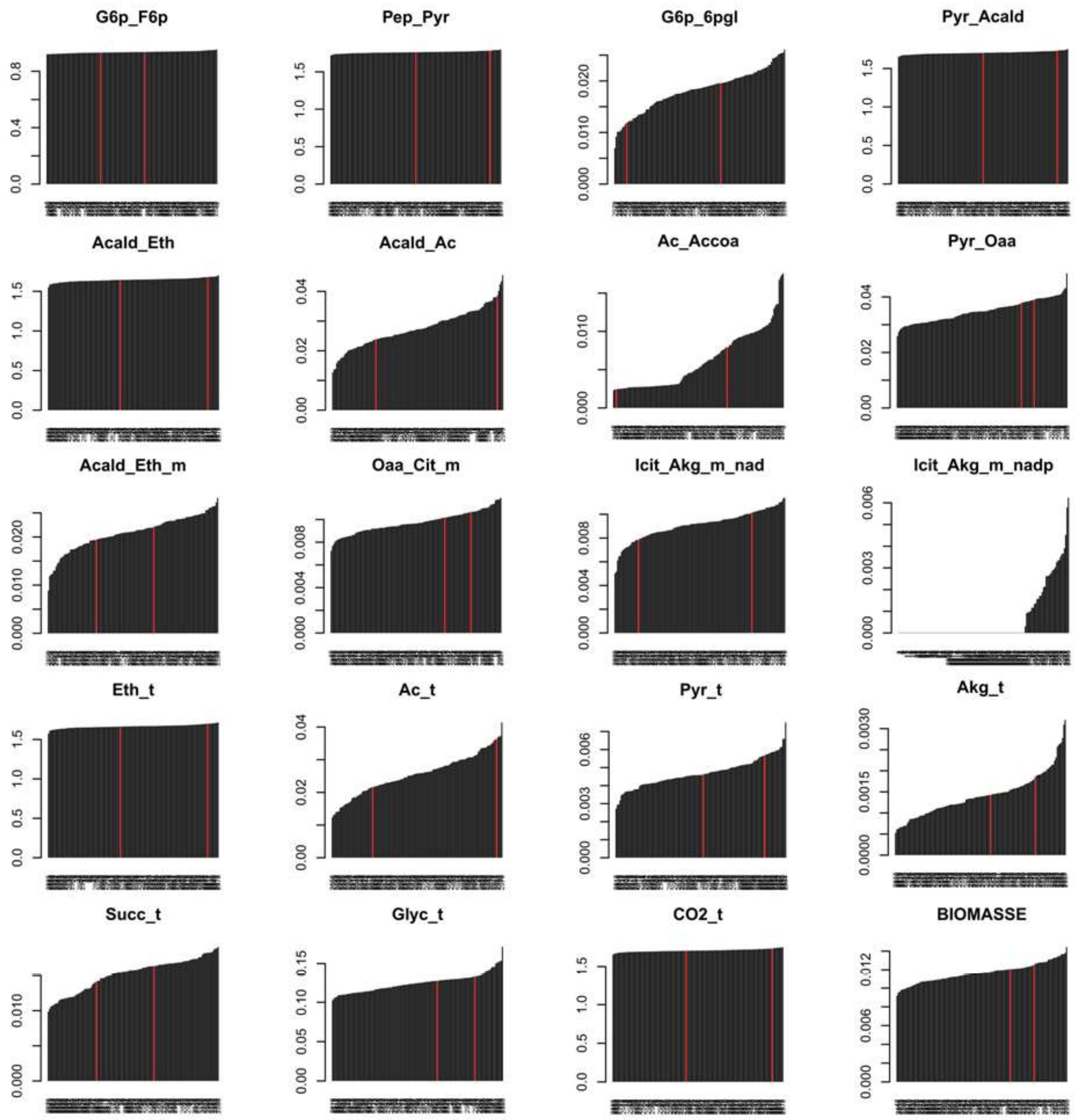

Supplementary Figure S2: Distributions of selected estimated CCM fluxes between the segregant strains and position of parent strains within the population (red lines).

Supplementary Table S4: Single fQTL mapping results.

Trait	Chromosome	QTL start [bp]	QTL peak [bp]	QTL end [bp]	LOD
Dhap_Glyc3p	II	662795	668586	701771	4.58
Glyc3p_Glyc	II	662795	668586	701771	4.58
Glyc_t	II	662795	668586	701771	4.58
Mal_tm	V	354177	385002	400836	4.01
Dhap_G3p	VII	40689	53880	58851	4.35
G3p_13dpg	VII	40689	53880	58851	4.37

13dpg_3pg	VII	40689	53880	58851	4.37
3pg_2pg	VII	40689	53880	58851	4.40
2pg_Pep	VII	40689	53880	58851	4.40
Pep_Pyr	VII	40689	53880	58851	4.41
Pyr_Acald	VII	40689	53880	58851	4.63
Acald_Eth	VII	40689	53880	58851	4.26
Oaa_Cit_m	VII	58851	76380	82449	4.05
Cit_Icit_m	VII	58851	76380	82449	4.05
Akg_Succoa_m	VII	52412	76380	82449	3.73
Succoa_Succ_m	VII	52412	76380	82449	3.73
Mal_Succ_tm	VII	52412	76380	82449	3.73
Akg_tm	VII	58851	76380	82449	3.87
Oaa_tm	VII	58851	76380	82449	4.05
Akg_t	VII	58851	76380	82449	3.87
Succ_t	VII	52412	76380	82449	3.73
ATP Shuttle	VII	52412	76380	82449	4.20
BIOMASS	VII	52412	76380	82449	3.73
Eth_tm	VIII	443664	466998	483121	3.45

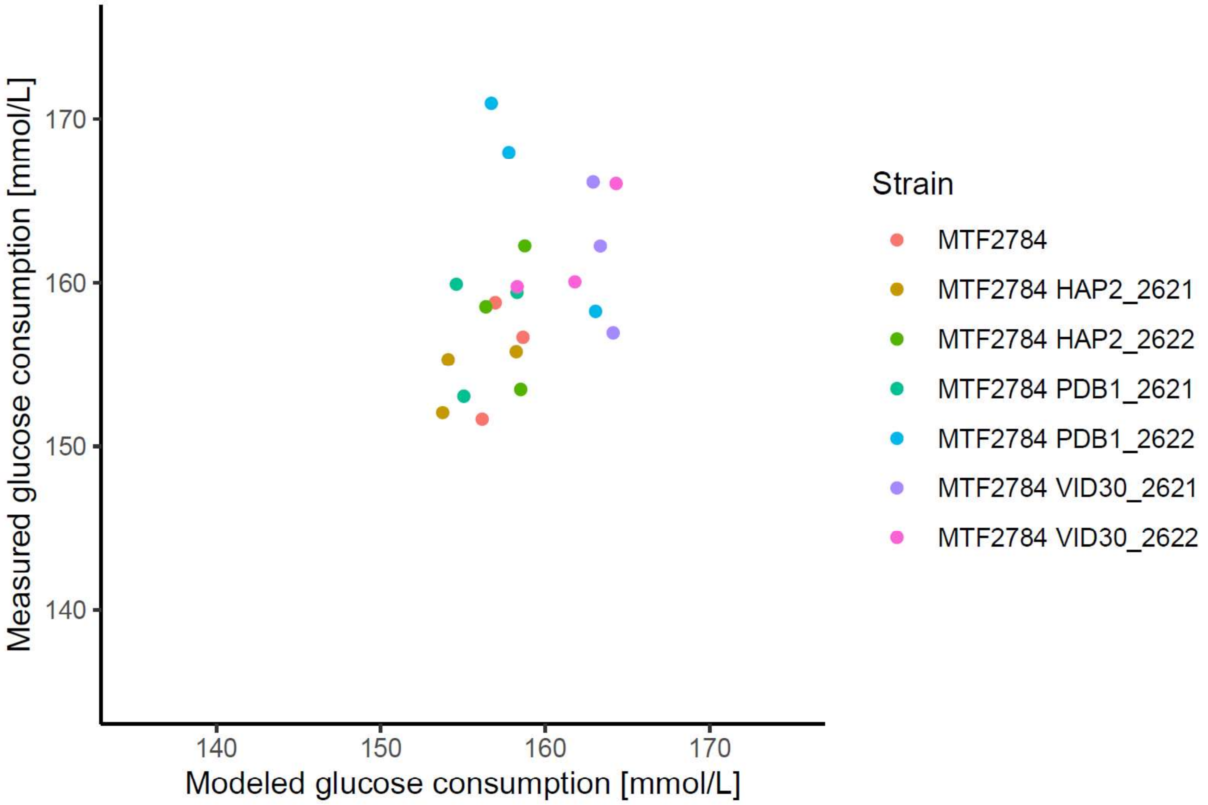

Supplementary Figure S3: Plot of experimental determined sugar consumption against predicted sugar consumption for the parental heterozygote (MTF2784) and evaluated homozygous candidate genes (in triplicates).

Supplementary Table S5: List of strains used for the phylogenetic analysis of target genes *PDB1* and *VID30*. Genomic sequences and descriptions of strain origin were obtained from the *Saccharomyces* Genome Database (SGD).

**Strain**	**Origin**
DBVPG6044	African
PW5	African
SK1	African
Y55	African
Y10	Asian
FostersB	beer
FostersO	beer
CLIB215	bread
YS9	bread
YJM339	clinical
YJM789	clinical
ZTW1	ethanol
BY4741	laboratory

BY4742	laboratory
CEN.PK	laboratory
D273-10B	laboratory
FL100	laboratory
FY1679	laboratory
JK9-3d	laboratory
S288C	laboratory
SEY6210	laboratory
W303	laboratory
X2180-1A	laboratory
YPH499	laboratory
T7	oak NA
YPS128	oak NA
YPS163	oak NA
CBS7960	rum
JAY291	rum
K11	sake
Kyokai7	sake
UC5	sake
EC9-8	soil
UWOPS05_217_3	soil
AWRI1631	wine
AWRI796	wine
BC187	wine
L1528	wine
LalvinQA23	wine
M22	wine
RedStar	wine
RM11-1a	wine
T73	wine
VL3	wine
YJM269	wine
EC1118	wine x flor
Vin13	wine x flor
